# Indoor Crowd 3D Localization in Big Buildings from Wi-Fi Access Anonymous Data

**DOI:** 10.3390/s19194211

**Published:** 2019-09-27

**Authors:** Anna Kamińska-Chuchmała, Manuel Graña

**Affiliations:** 1 Faculty of Computer Science and Management, Wroclaw University of Science and Technology, 50-370 Wrocław, Poland; 2 Computational Intelligence Group, Computer Science Faculty, University of the Basque Country, UPV/EHU, 00685 San Sebastián, Spain; manuel.grana@ehu.es

**Keywords:** remote sensing, indoor crowd detection, geostatistical methods, Wi-Fi sensors, wireless sensor network

## Abstract

Indoor crowd localization and counting in big public buildings pose problems of infrastructure deployment, signal processing, and privacy. Conventional approaches based on optical cameras, either in the visible or infrared range, received signal strength in wireless networks, sound or chemical sensing in sensor networks need careful calibration, noise removal, and sophisticated data processing to achieve results in limited scenarios. Moreover, personal data protection is a growing concern, so that detection methods that preserve the privacy of people are highly desirable. The aim of this paper is to provide a technique that may generate estimations of the localization of people in a big public building using anonymous data from already-deployed Wi-Fi infrastructure. We present a method applying geostatistical techniques to the access data acquired from Access Points (AP) in an open Wi-Fi network. Specifically, only the time series of the number of accesses *per* AP is required. Geostatistical methods produce a 3D high-quality spatial distribution representation of the people inside the building based on the interaction of their mobile devices with the APs. We report encouraging results obtained from data acquired at a building of Wroclaw University of Science and Technology.

## 1. Introduction

Crowd detection is an important security problem, which is further complicated in European countries by the increasing public sensitivity about personal data protection that has led to new regulations such as the European General Data Protection Regulation (GDPR) [1] imposing tight controls and limitations to the collection and processing of personal data [2]. Therefore, crowd detection tools that ensure personal data privacy by relying only on anonymous information are highly desirable. In this paper, we propose an alternative solution to indoor crowd localization in big buildings that uses existing Wi-Fi Access Points (APs) of an open wireless network deployed in the building. The approach is completely anonymous because only the number of people (NoP) accessing the network is recorded, and no personal data is captured in any way. The activity recorded at each AP is used to build up a spatial model of the positions of the people conditioned to the number of users detected at each AP location. We use geostatistical methods to make dense spatial estimations of the localization of people inside the building. The approach is based on the rather natural assumption that people will be carrying their smartphones or another Wi-Fi-enabled device. The estimation process does not need any training phase, so that it works smoothly when the APs are moved in the space or some new APs are added to the system. The only requirement is to have updated knowledge of the actual spatial localization of the APs. Intuitively, our proposed system uses the Wi-Fi-APs as a network of sensors, so that the geostatistical methods are the computational sensor probes in the space inside the building. In this introductory section we will first do a short review of most relevant crowd detection and counting approaches found in the literature in order to set the stage for our proposal. Secondly, we provide a description of the system, setting the computational background. Finally, we close the introductory section with the detail of the paper remaining content.

### 1.1. Crowd Detection Approaches

Up to now, most works on crowd counting, detection, and anomaly behavior analysis are supported on optical data sources [3,4,5,6,7], which capture a great deal of personal information. Besides privacy issues, optical sensors, which are quite effective in open spaces, are hindered in indoor settings by many occluding architectonic elements. Some alternative approaches for indoor crowd detection/counting rely on passive acoustic and chemical sensors [8], or perturbations in the propagation medium of the Wi-Fi signal [9]. The success and replicability of such systems are limited by the range of the sensors, as well as by the noisy nature of their produced signals. Thus, indoor crowd counting/detection respecting privacy remains a challenge.

#### 1.1.1. General Artificial Vision Approach

The study of crowd behavior is a relevant topic in many areas, ranging from security to urban planning [10]. For instance, early works tried to estimate the density of the crowd in a subway platform [11]. Most of the reported works are based on computer vision methods applied to optical sensors (cameras). Crowd detection is not a problem *per se* in this setting: systems carry out the visual detection of individual persons and their dynamics. Crowd behavior analysis is defined on the aggregation of individual behaviors. Computational methods whose application has been already reported [5] include classical background subtraction, modeling of moving persons by optical flow computation [12], image texture analysis [13], dynamic modeling by hidden Markov models [10], hierarchical Dirichlet processes [14], reversible jump Markov chain Monte Carlo sampling [15], markerless tracking [16], and, recently, applications of deep learning neural network architectures [17,18,19]. Most of the works found in the literature refer to outdoor scenes, where the focus is on the detection of anomalous behavior characterized according to collectiveness, stability, conflictivity, and uniformity of behaviors. Machine learning approaches need labeled data for training and validation. Thus, synthetic labeled crowd video generation has been proposed [20] with some success. A physical modeling approach proposed by [21] computes the energy-level distribution of a particle kinetic model of the crowd in video streams. Crowd abnormal behavior is detected based on changes in this crowd energy level.

#### 1.1.2. Optical Anonymous Sensors

The protection of privacy and private data is a growing concern in European countries. Thermal infrared cameras offer good detection capabilities and anonymity [22], with some straightforward applications such as fever detection in indoor crowds [23]. They were proposed in the early research works on outdoor human crowd behavior monitoring [24] in conjunction with optical cameras in the visible range and radar sensors (for the detection of guns and metal weapons). Thermal imaging is also robust to imaging conditions such as fog, or low illumination allowing for detection of specific threats in crowded scenarios [25]. Moreover, its greater contrast allows easier and more robust crowd density estimation [26]. However, infrared and visible spectrum cameras can only perceive what falls in their line of sight [6]. Instead of thermal imaging, ref. [27] uses laser range scanners to carry out person detection. This approach is robust to illumination conditions, but it is very sensitive to occlusions so that a specific crowd density estimation must be developed. The authors also propose the analysis of social media posts to predict likely event locations, which is needed for the deployment of the laser range sensors.

#### 1.1.3. Sensor Networks

Recently, there has been growing interest in Wireless Sensor Networks (WSNs) as a remote sensing tool for crowd detection and crowd size measurement. A combination of sensors for carbon dioxide concentration, sound intensity level, and received signal strength deployed in a network of motes in strategic areas was considered in [8]. Such signals are inherently anonymous, but they are also very noisy and need careful processing. The work in [8] focuses on careful spatio-temporal clustering techniques to achieve some degree of detection robustness using such sensors. In a different approach, using the received signal strength indicator (RSSI) to estimate the distance from the mote carried by the person to the beacon broadcasting its GPS information, ref. [28] provides a robust algorithm for the localization of the individuals carrying the motes. Such localization information may be used for crowd size estimation and localization, but it requires the distribution of the motes among the public. This could be achieved in specific events, but this mote deployment is unlikely in general scenarios.

#### 1.1.4. Wi-Fi Based Approaches

The pervasiveness of Wi-Fi networking devices has encouraged the use of this infrastructure for indoor crowd detection and counting. Some approaches use the physical properties of the signal to achieve crowd counting. For instance, ref. [29] captures the channel state information (CSI) by means of a modified firmware, which contains information about the signal propagation medium, and, consequently, can be used for crowd size estimation. The CSI signal is subject noise removal by wavelet decomposition, and then computing various feature extraction procedures on the cleaned signal. Finally, support vector machines (SVM) are trained to provide crowd size predictions. The approach is inherently limited to small spaces due to the low spatial resolution of CSI, but it is inherently anonymous and does not need any additional device, though reproducibility is conditioned to the availability of the modified firmware installation, which poses additional security and reliability concerns. A similar approach [9] uses the RSSI values of a grid of wireless emitting/receiving sensors deployed in an otherwise empty room. The RSSI of the nodes in the grid is calibrated without people interference, and the difference of RSSI when some people enter and move around is measured. Clustering is used to create a rough map of the RSSI values into crowd density values. A different signal processing approach is based on the Doppler spectrum of the signal at the Wi-Fi receiving station, which is changing according to the number of people in the room, so that a classifier can be trained to estimate the number of people based on the Doppler spectrum [30]. Notice that in all these approaches the people in the room is not using the Wi-Fi network for the purposes of the experiment. Their single role is interfering the RF signal propagation. Therefore, they are not readily comparable with our approach as described in next subsection. Furthermore, these approaches cannot be extrapolated to big buildings with signals having to cross walls and furniture. In contrast, the approach proposed in this paper does not need new infrastructure deployment and works for large buildings regardless of conditions such as furniture and walls. The experiment reported in [2] combines several estimation approaches, namely RSSI and position-based approaches, in an open space event. Their use of the spatial localization of the Wi-Fi-APs is a nice antecedent of our own approach.

### 1.2. Description of Proposed System

**Figure 1:**
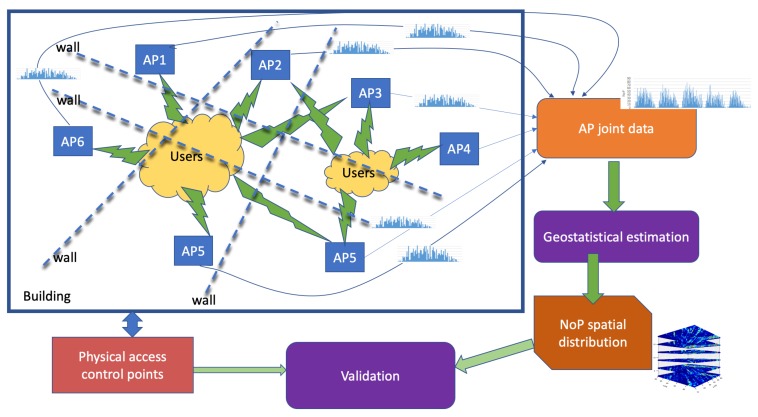
Diagram of the proposed system.

Figure 1 contains a diagrammatic representation of the proposed system. People is located inside a building where a Wi-Fi service is deployed. Each person is assumed to have an active Wi-Fi enabled device that he/she will be trying to connect to the Wi-Fi service. In the case of the experimental data recording described below, the Wi-Fi is open to all users, so it is highly likely that the devices will be effectively connected. In other cases, the system may be recording the access attempts. In any case, we have for each Wi-Fi AP a record of the number of users interacting with the network. This interaction uses the MAC identification of the device; however, we are not tracking individual devices, so we do not need/use MAC identification information. We collect the AP information about the number of accesses to a central server, which collects their time series. This server could carry out some data cleaning, such as removing laser printers and other Wi-Fi-enabled devices that are not personal [2]. In our data gathering example, it carries out the averaging *per* hour, which is our time resolution for the reported computational experiments. As represented in the figure, the people in the building may be separated by walls, and, depending on their actual position, they can be alternating connection between APs. Specifically, we remark that people are not necessarily in the same room with the Wi-Fi receiver to stress that we are not modeling the signal propagation in any way. The data from the APs (including their spatial localization in the building) is used by a Geostatistical estimation system to compute an estimation of the spatial distribution of the number of people (NoP) for the specified time interval. For validation purposes we use the information provided by some physical access control points about the number of people crossing them. Specifically, some staff offices are endowed with smart card access, which has been used to obtain ground truth validation data.

#### 1.2.1. Computational Methods

Geostatistical methods [31,32] have been used to achieve spatial estimation in many domains such as geology, mining, ecology, epidemiology, nuclear safety, finance, energy management, etc. For instance we have been working on spatial electric load forecasting [33], the discovery of client-perceived web performance [34], and spatial models for efficiency prediction of Wi-Fi networks [35]. The big advantage of geostatistical methods is their proven ability to generate spatial estimations of the value of the variable considered using local measurements. In short, geostatistical methods first carry out the estimation of the spatial covariance functions modeling the spatial distribution of the variable of interest. Then, they use these covariance estimations in order to generate samples of the variable of interest over a spatial grid. Data acquired at specific spatial locations are used to formulate the linear equations whose solutions provide the spatial predictions of the variable of interest according to a process called kriging. In our work reported in this paper, the variable of interest is the number of people (NoP) at each spatial grid cell, and the local measurements are the NoP trying to access each Wi-Fi-AP. To the best of our knowledge, no previous works have applied geostatistical methods to crowd detection/counting.

#### 1.2.2. Intuitive Description of Our Approach

The Wi-Fi-APs record the number of people (NoP) connected to them. These readings are partial observations of the crowd(s) inside the building taken at their specific spatial locations. From these observations we can build an estimation of the localization of the people inside the building. For this estimation we do not use any feature of the RF signal arriving at the AP, we only use the NoP connected to the AP at each time interval of interest. From these NoP time series we derive the spatial covariance information, i.e., how the NoP value at a specific spatial location is related to the NoP at another spatial location. The spatial covariance can be used to generate the expected values of NoP in locations where we do not have sensors providing us direct information. In other words, spatial covariance function is used to fill the holes in our data, which are almost all the space of interest because we have a few APs. For this estimation process we consider the space discretized into cells, where we want to guess the NoP value. For this process of “filling the holes” we use a very specific mathematical tool, called Turning Bands (TB) [36] which consists of drawing lines in the space and carrying out the estimation of NoP values along each line, instead of directly trying to generate the NoP values in the high-dimensional (3D) space. This approach is much more economical in computational cost. Because the TB method is originally defined for Gaussian processes, it is necessary to carry out a previous transformation of the data to obtain Gaussian distributed data (anamorphosis). Finally, we use the actual data recorded at the APs to fit our spatial model to the actual readings. This process called kriging consists of the resolution of the linear equations relating the APs NoP measurements to the model. We seek to minimize the prediction error at the spatial locations of the APs, thus correcting the entire spatial estimation of the values of NoP.

#### 1.2.3. Software Resources

The computational experiments reported in this paper were performed using R language and RStudio version 1.1.447, which is available as Free Software under GNU license [37]. Specifically, to carry out the geostatistical estimation processes for spatial predictions, we make use of the RGeostats package developed by the Geostatistical Team of the Geosciences Research Center of MINES ParisTech [38].

### 1.3. Paper Content

The paper contents are as follows: Section 2 presents the data gathering environment. Section 3 describes the computational details of the geostatistical approach used for crowd localization. Section 4 presents the achieved spatial distribution estimation results for the given experimental data. Section 5 contains a discussion of results and several aspects of the proposed system. Finally, Section 6 gives some conclusions and lines for future research.

## 2. Experimental Data Gathering

**Figure 2:**
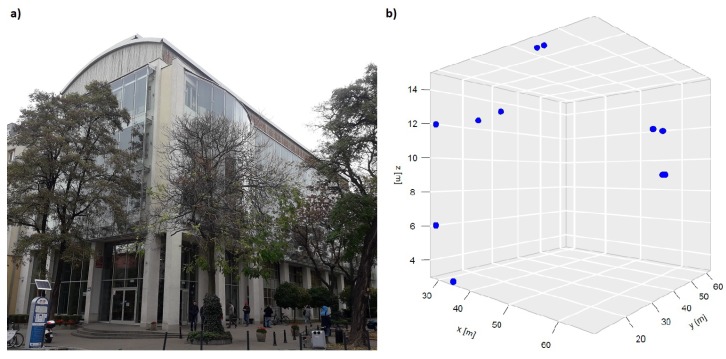
(**a**) Building B-4 located in WUST main campus in Wroclaw, Poland. (**b**) Localization of Wi-Fi access points (APs) in B4 building.

The experimental data used in this paper are the open wireless network access measures obtained by the informatics support services in a building of the Wroclaw University of Science and Technology (WUST) campus. Specifically, we have the recording of the number of people connected to each of the access points (APs) in the building B-4 in the main academic WUST campus provided by the informatics and communication support services. This building hosts the Dean offices of two schools, a few lecture halls, research laboratories, two libraries, and administrative and researchers’ offices. People staying in this building are predominantly students and WUST employees, such as lecturers or administrative workers. There are 11 APs in the five-story building. We also consider the ground floor so that we have six planes in our visualizations. Wi-Fi-AP locations in B-4 building floors are as follows: one on the first, one on the second, two on the third, five on the fourth, and two on the fifth. A visualization of Wi-Fi APs localization is presented in Figure 2b. Our goal is to compute estimations of the spatial (3D) distribution of the values of variable “Number of People (NoP)” to achieve crowd localization on the basis of the NoP recorded at each of the APs. The Wi-Fi network APs are therefore used as a kind of Wireless Sensor Network (WSN). All APs use frequency standards: 2.4 GHz in IEEE 802.11b/g/n and 5 GHz in IEEE 802.11a/n. The APs have 6 or 4 antennas, depending on the model. In AP models with six antennas there are three antennas working in the 2.4 GHz frequency and three antennas working at 5 GHz, while four antenna models have DualBand antennas.

**Figure 3:**
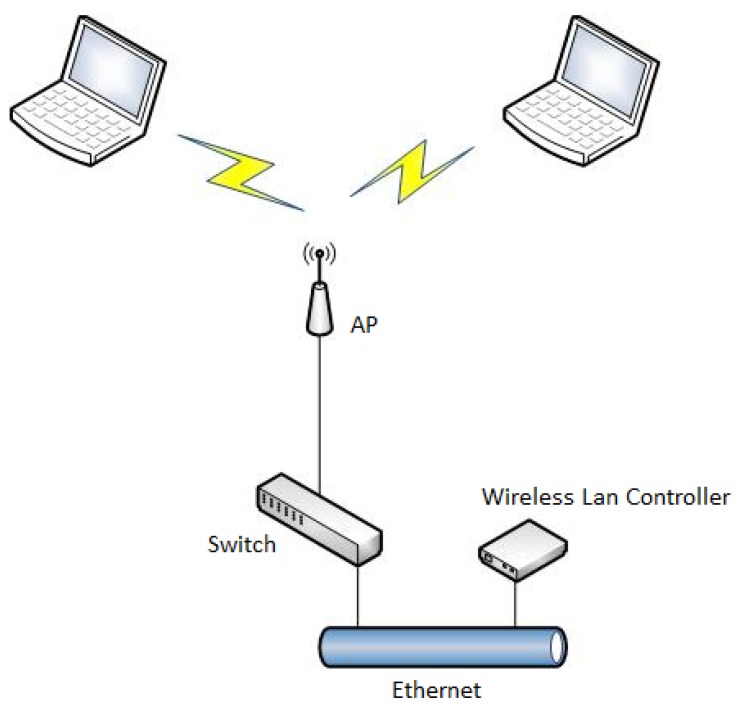
System diagram of the AP connection in WUST network.

**Figure 4:**
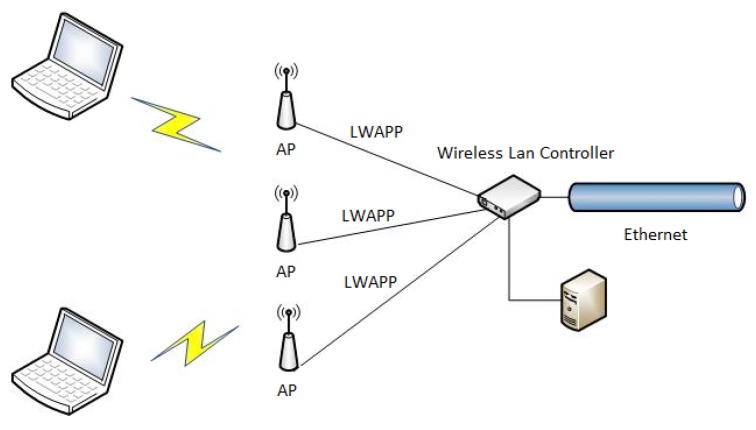
Model of a centralized system using the LWAPP protocol.

Figure 3 shows the diagram of the AP wired connection to the central Wi-Fi controller as deployed in the WUST network following a star topology as shown in Figure 4. The wireless clients and the Ethernet are not in the same virtual local area network (VLAN). All APs use the lightweight access point protocol (LWAPP) for communication with the controller. In wireless local area networks (WLAN), managing individual APs becomes more troublesome when the number of APs increase. Individual AP configuration is time consuming, and may cause security compromising inconsistencies such as the use of the same RF channels by neighboring APs, creating undesired collisions and loss of information. The centralized WLAN control system implemented by the LWAPP protocol ensures uniform management of security policies, Quality of Service (QoS), radio channel assignments, and it also enables network smooth incremental development, automatically configuring the newly added devices. In the LWAPP all communication between the AP and the management platform (controller) can be routed through two different channels: the LWAPP control channel and the LWAPP data channel responsible for data encapsulation. Its task is to “transfer” most of the functionalities (e.g., QoS policy, bandwidth management, security policy, RRM) from the AP to the central management unit using Split-MAC technology. Another feature of the LWAPP protocol is the fact that only the control channel is subject to the encryption process (AES). Unlike traditional WLAN networks, networks built based on LWAPP can independently manage radio channels and dynamically allocate them, in order to have a centralized interface for policy management, security, QoS, and appropriate assignment of clients to VLANs. It is, therefore, possible to record the NoP at each AP from the statistics available at the central controller, without the need to know the MAC or any other identificatory information of the wireless clients. In other words, we may count the number of client data virtual tunnels through the central controller without knowing the actual content of the data frames to obtain the NoP value at each instant. Furthermore, the count is independent of the spatial distribution of the devices because it is done at the centralized controller. As a kind of corollary, we are not influenced by conditions of the physical process of wave propagation.

**Figure 5:**
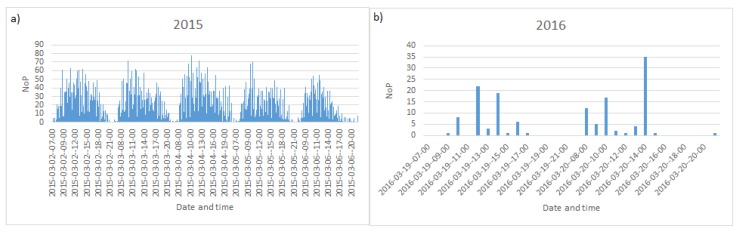
Number of People (NoP) served by all APs in B4 building: (**a**) during workdays between 2 and 6 March 2015, (**b**) during the weekend between 19 and 20 March 2016.

**Figure 6:**
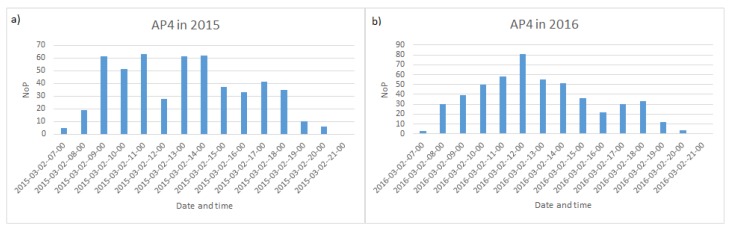
Number of People (NoP) served by AP4 in building B-4: (**a**) 2 March 2015 (Monday), (**b**) 2 March 2016 (Wednesday).

The data available for the realization of the computational experiments are the NoP averaged values at each AP recorded every hour between 7:00 a.m. and 9:00 p.m. in the period between 1st and 31st of March of years 2015 and 2016. Figure 5a shows the NoP during workdays of the first week of March 2015 and—for contrast—Figure 5b plots NoP during a weekend in March 2016. Figure 5a reflects the periodical behavior of the overall people in the building during the week, related to the fact that classes start at 7:30 a.m. and finish at 8:30 p.m. Generally, during the day, classes start quarter after an odd hour. Figure 5b shows that there are much fewer students and administrative workers during the weekend and the lack of periodicity due to class schedules. Figure 6 plots the number of people detected by the same AP in March 2nd of 2015 and 2016, thus illustrating the difference introduced by the passing of the time. The NoP in this AP is higher in 2016, as a result of the increasing use of mobile computing platforms, such as mobile phones, tablets on top of personal laptops with a wireless connection.

**Figure 7:**
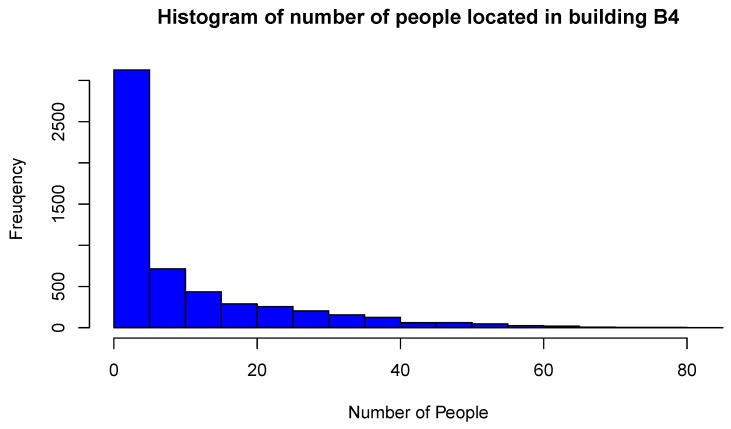
Histogram of global NoP inside building B-4 in 2015 measuring period.

**Figure 8:**
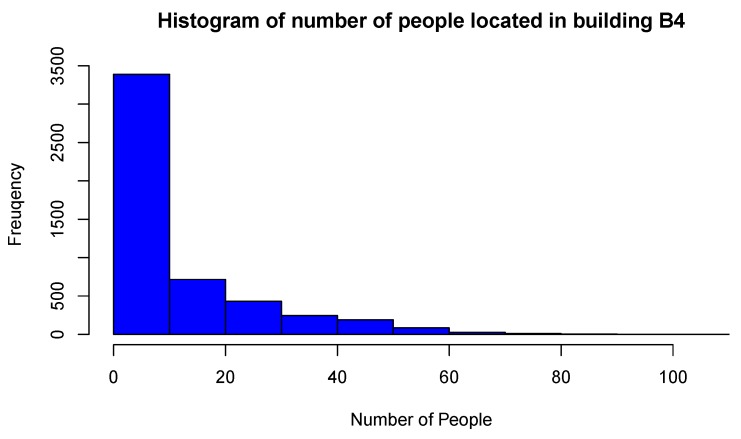
Histogram of global NoP inside building B-4 in 2016 measuring period.

The basic statistics of variable NoP recorded in building B-4 are presented in Table 1. The maximum number of people inside the building is 81 in 2015 and 102 in 2016, thus confirming the growing trend of the use of Wi-Fi enabled devices and building occupancy between these years. Figure 7 and Figure 8 show the histograms of the global NoP variable in 2015 and 2016, respectively. Frequency reflects hourly NoP values recorded from all APs during the whole year. The highest frequency bin of people is in the range [0, 5] in 2015, and [0, 10] in 2016. For both years the histograms are extremely left-skewed. A wider range of bins and higher histogram maximum in 2016 is another indication of increasing NoP in building B-4 across years, and greater variability of their spatial localization.

## 3. Spatial Estimation Mathematical Methods

Geostatistical methods [31,32] have been developed to compute spatial predictions of variables in the mining and oil industries, where, for instance, there is a strong interest in predicting the location of oil deposits on the basis of a reduced number of measurements. Strictly speaking, geostatistical methods are distribution-based sampling methods for spatial random variables [31]. Therefore, specialized literature refers to the computations involved as simulations. However, simulation outputs are used as spatial predictions for all practical purposes in many domains such as geology, ecology, epidemiology, nuclear safety, finance, and energy management. As the general meaning of prediction is temporal instead of spatial, we will use the term “estimation” to avoid confusion with time series prediction. A widely used geostatistics computational approach is the Turning Bands (TB) method [31,36,39,40,41,42]. We will use the TB method to generate estimations of NoP values in the 3D discrete cells corresponding to the five stories and ground floor of the target building. In essence, the TB method reduces a multidimensional variable estimation problem to a one-dimensional one.

In an intuitive description, our goal is to compute a NoP value for each of the spatial cells discretizing the space of interest (the floors of the building) with knowledge of a perceived value of NoP in some of them (corresponding to APs locations). In other words, we want to fill the holes in the occupancy map of the building in a way that is concordant with the observations gathered at the cells containing an AP. From the AP data we may compute the variograms that give us information about the spatial covariance functions. This information is used in the so-called unconditional simulation to generate estimations of the expected NoP values at each spatial cell by the TB method. The TB is based on the idea of sampling the high-dimensional space (3D) by several lines that crosses it densely. It is easier and faster to generate estimations along a line than in the entire space of interest. The integration of the estimations produced by the diverse lines that meet at each cell gives us the 3D estimate at this cell. The conditional simulation fits the unconditional results to the actual observations obtained at the APs, correcting the spatial estimation by maximization of the likelihood of these observations. This is achieved by a kriging process that solves the linear equations relating the APs measurements and the unconditional simulation. The data is assumed to be Gaussian distributed, therefore a step of transformation of the data to obtain Gaussian distributed data (anamorphosis) is required, as well as the reverse transformation of the results to follow the original probability distribution.

Formally, the TB method averages a large number of independent estimations along random lines scanning the *m*-dimensional space. The value of the predicted variable at point x of the *m*-dimensional space is given by the sum of the values at point x of the *n* line stochastic processes. Formally, the estimation of the spatial process value at location x is defined by:

(1) $$Y^{n}\left(x\right)=\frac{1}{\sqrt{n}}\sum_{k=1}^{n}X_{k}\left(<x,\theta_{k}>\right),x\in R^{m}$$

where $\left(\theta_{k},k\in N\right)$ is a collection of random directions densely covering a hemisphere of dimension *m*, $S_{m}^{+}$, and $\left(X_{k},k\in N\right)$ is a sequence of independent stochastic processes defined on a line, each with covariance $C_{\theta_{k}}$. The overall spatial process covariance is defined as:

(2) $$C_{m}\left(h\right)=\frac{1}{n}\sum_{k=1}^{n}C_{\theta_{k}}\left(<h,\theta_{k}>\right)\;h\in R^{m}.$$

**Figure 9:**
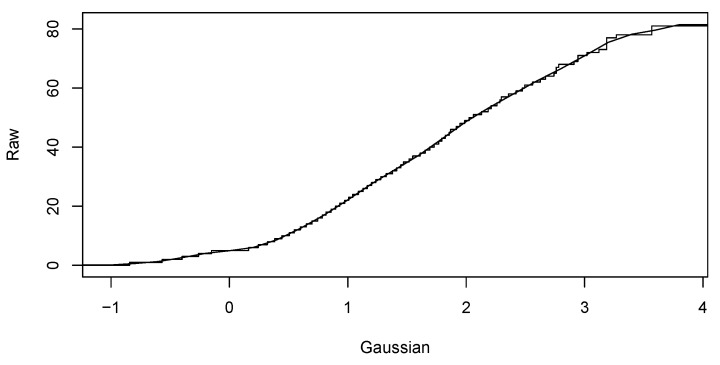
The Gaussian anamorphosis of the NoP process data in March 2015.

**Figure 10:**
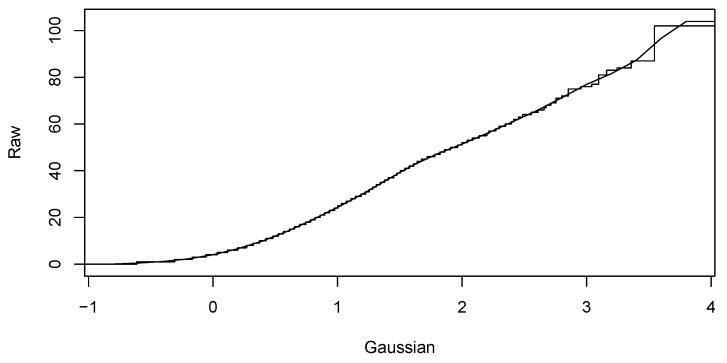
The Gaussian anamorphosis of NoP process data in March 2016.

**Figure 11:**
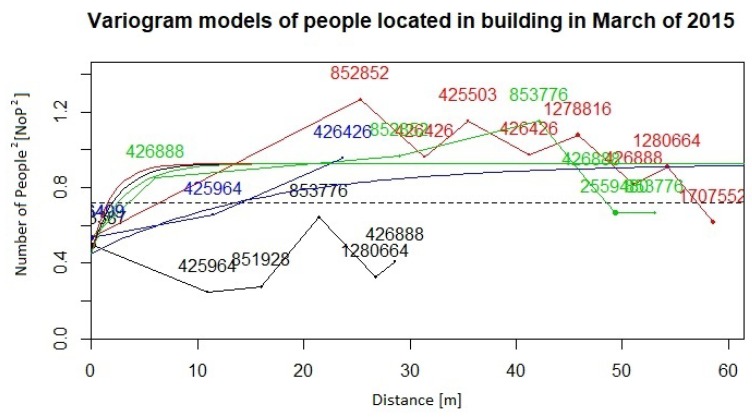
Empirical variograms and fitted exponential variogram models (smooth curves) computed in four directions on 2015 NoP values after Gaussian anamorphosis (numbers indicated at nodes of the empirical variogram functions meaning the number of pairs of NoP).

**Figure 12:**
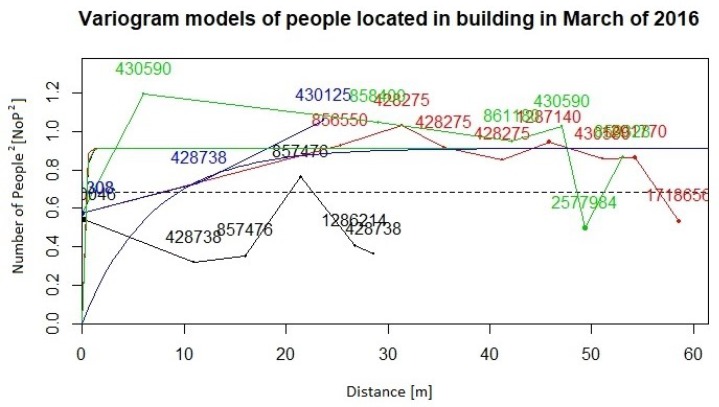
Empirical variograms (jagged lines) and fitted exponential variogram models (smooth curves) computed in four directions on 2016 NoP values after Gaussian anamorphosis (numbers indicated at nodes of the empirical variogram functions meaning the number of pairs of NoP).

Our sequence of computational steps is as follows:

1. Preparation of the database according to input restrictions.
2. Calculating fundamental statistics and assessing the Gaussianity of the input data.
3. Computing Gaussian anamorphosis on the raw data [43,44] to transform the input data into Gaussian distributed data. Computations were carried out using 100 Hermite polynomials for each year. The upper interval for 2016 indicates a wider expansion of the raw data. Finally, the anamorphosis result will be used in the random diagram of the Gaussian curve to obtain a random graph of the original variable. Graphs of Gaussian anamorphosis for the data collected each year are presented in Figure 9 and Figure 10, respectively.
4. Computing the empirical variogram function in given direction and parameters.
5. Approximate the variogram function by fitting a parametric model for their use in the kriging conditional estimation. For both data collections from the years 2015 and 2016, we compute empirical variograms over four directions: 0, 45, 90, 135 degrees. Distance lags in each direction are 5, and the number of lags for all directions is 15. The maximum distance used for the estimation of the empirical variogram functions was 60 m for the data from both years. Afterwards, variogram functions were approximated by the exponential and spherical models. Figure 11 and Figure 12 present the empirical variograms and fitted exponential models at the four directions for the years 2015 and 2016, respectively.
6. Choose appropriate parameters of TB and the number of repetitions *per* model.
7. Run TB algorithm to compute spatial estimations of the variable of interest, $Z\left(x\right)$, specifically in our study the values of the variable NoP at spatial location x.

**Figure 13:**
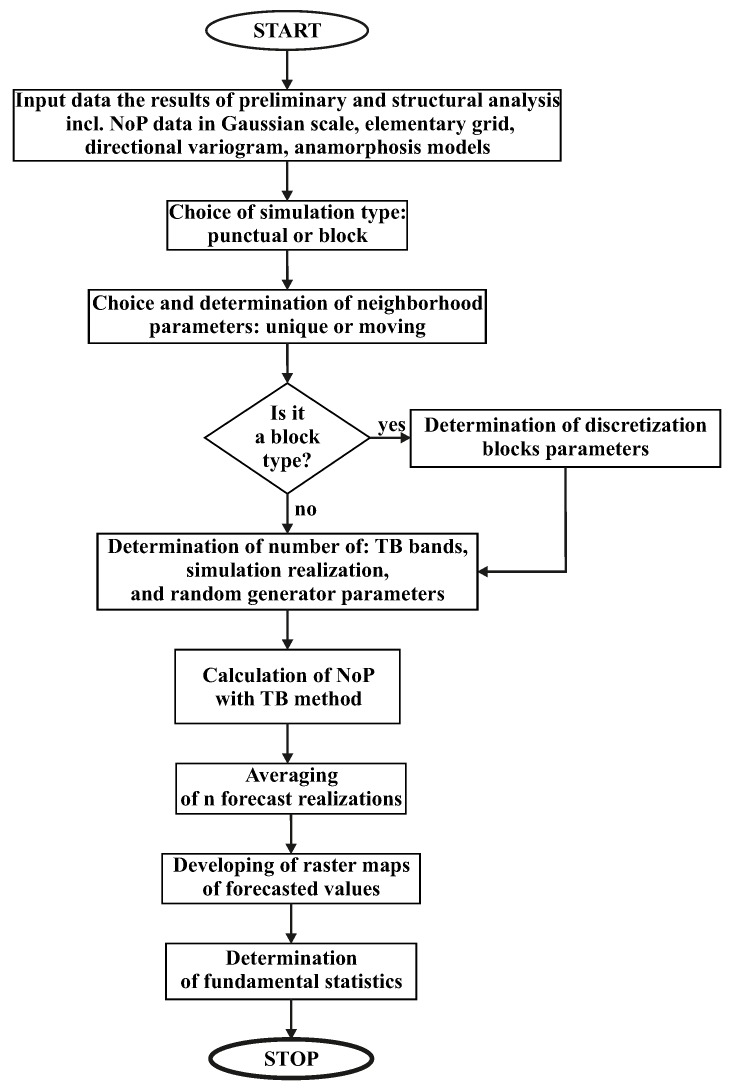
Block diagram TB spatial estimation process.

**Figure 14:**
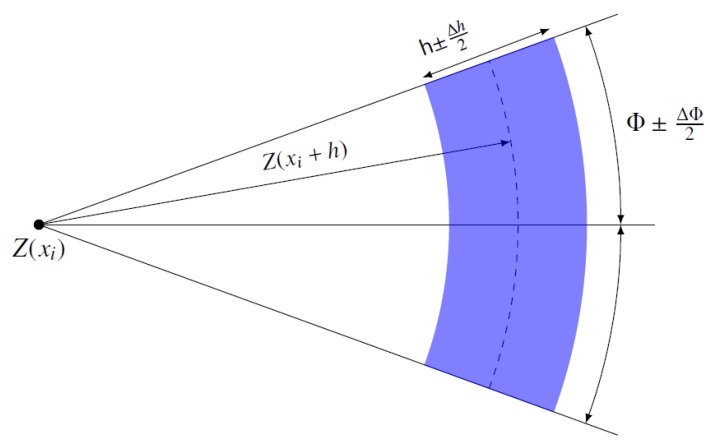
Tolerance of the directional variogram as a function of distance h.

**Figure 15:**
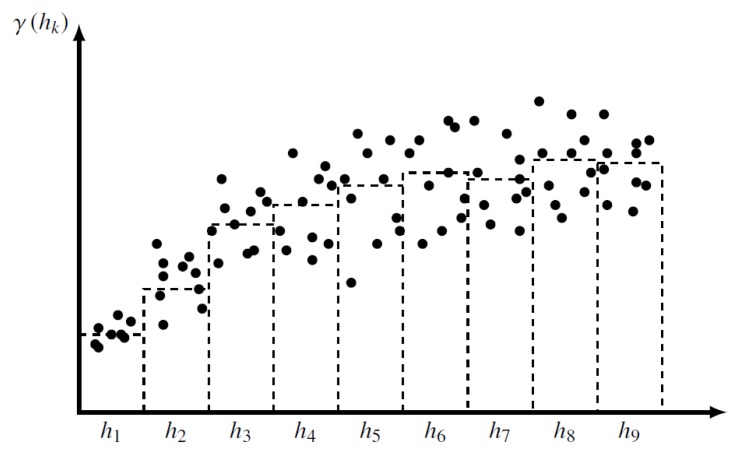
The directional variogram obtained by averaging the dissimilarities $\gamma^{*}$ for given classes.

**Figure 16:**
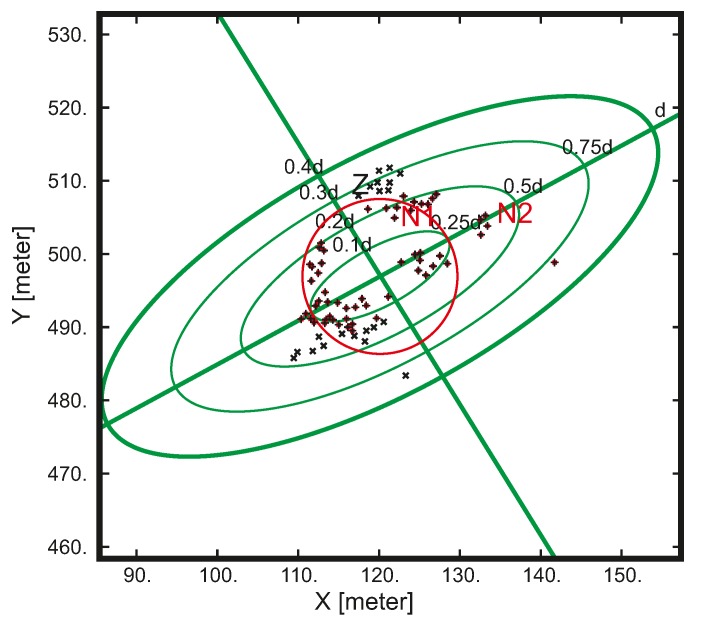
Sample moving neighborhoods. Green ellipses define the anisotropic neighbourhoods for increasing threshold distances. Red circle shows an isotropic neighbourhood for comparison. Points N1 and N2 belong to the 0.5d neighborhood while Point Z does not.

A block diagram presented in Figure 13 provides a graphical representation of the process. In the following, we provide a formal description of the computational steps of the TB algorithm:

1. Select directions $\theta_{1},\ldots ,\theta_{n}$ in *m*-dimensional space so that $\frac{1}{n}\sum_{k=1}^{n}\delta_{\theta_{k}}\approx \varpi$.
  The covariance of the spatial process $C_{m}$ is obtained by summing the projection of the process realizations for a given number of lines of covariance $C_{\theta_{k}}$.
2. Generate realizations of standard, independent 1D stochastic processes $X_{1},\ldots ,X_{n}$ with covariance functions $C_{\theta_{1}},\ldots ,C_{\theta_{n}}$.
  When all the $C_{\theta_{k}}$ are identical to a covariance function, denoted $C_{1}$, the relationship between $C_{1}$ and $C_{m}$ is as follows [42]:
  (3) $$C_{m}\left(r\right)=2\frac{\left(m-1\right)\omega_{m-1}}{m\omega_{m}}\int_{0}^{1}{\left(1-t^{2}\right)}^{\frac{d-3}{2}}C_{1}\left(tr\right)dt$$
  where: $\omega_{m}$ stands for the volume of the unit ball in *m*-dimensional space. In our case, m=3, it reduces to:
  (4) $$C_{3}\left(r\right)=\int_{0}^{1}C_{1}\left(tr\right)dt$$
  or equivalently:
  (5) $$C_{1}\left(r\right)=\frac{d}{dr}\left(rC_{3}\left(r\right)\right)$$
  Knowing variogram and variance *C(0)* for a stationary random function *Z* is tantamount to knowing its covariance. To determine a covariance function for each variable, we compute a variogram function, which is defined for any displacement vector h as:
  (6) $$\gamma \left(h\right)=\frac{1}{2}E\left({\left(Z\left(x+h\right)-Z\left(x\right)\right)}^{2}\right).$$
  The tolerance of the directional variogram as a function of distance h is illustrated in Figure 14.
  The directional variogram is obtained by averaging the local estimations $\gamma^{*}$ for given line processes, as shown in Figure 15.
3. Compute $Y^{n}\left(x\right)=\frac{1}{\sqrt{n}}\sum_{k=1}^{n}X_{k}\left(<x,\theta_{k}>\right)$ for each x∈D, where D is the set of locations of the discretized domain where we generate the process value estimations.
  There is a wide variety of sampling methods for stochastic processes with a given covariance function $C_{1}$. The most popular are the spectral, dilution, and migration methods.
4. Compute the kriged estimate $y^{*}\left(x\right)=\sum_{i=1}^{S}\lambda_{i}\left(x\right)y\left(c_{i}\right)$ for each x∈D.
  Let *Y* be a stationary Gaussian random function with average value 0, variance equal to 1, and covariance function *C*. The goal is to generate a sample of *Y* satisfying the contour conditions given be the known values $Y\left(c_{i}\right)=y\left(c_{i}\right)$ at specific spatial locations ${\left\{c_{i}\in R^{m}\right\}}_{i=1}^{S}$. Kriging involves the resolution of a system of linear equations, requiring the definition of a set of neighborhood points. There are two types of neighborhood: unique and moving. A unique neighborhood needs only to compute once the inverse kriging matrix because it remains the same for all considered points. Thus, solutions using unique neighborhood can be computed much more quickly than moving neighborhood. When using a moving neighborhood, the closest points located in a sphere or ellipsoid are selected to be correlated with the considered point x. Examples of moving neighborhood are shown in Figure 16. The moving neighborhood search in our study has been performed by angular sectors and the neighborhood ellipsoid is anisotropic in 3D.
  The neighborhood points are produced by an algorithm which takes into account criteria such as rotation, search ellipsoid (it defines the maximum distance along the main axes *U*, *V*, *W* after rotation), anisotropy, the minimum number of points considered in the range search, etc. If the neighborhood is anisotropic, the search ellipsoid is defined by:
  (7) $$d=\sqrt{{\left(a_{u}d_{u}\right)}^{2}+{\left(a_{v}d_{v}\right)}^{2}+{\left(a_{w}d_{w}\right)}^{2}},$$
  where $d_{u}$, $d_{v}$, and $d_{w}$ correspond with distance along the axis of the new coordinate system, and $a_{u}$, $a_{v}$, and $a_{w}$ correspond to the ratio between the maximum distance upon axis *U*, *V*, *W* and the maximum distance $d_{max}$, respectively.
5. Generate samples of a Gaussian random function with mean 0, and covariance *C* in domain D conditioned to the contour points.
  Obtained values are: $\left\{z(x),x\in D\right\}$ and ${\left\{z(c_{i})\right\}}_{i=1}^{S}$.
6. Calculate the kriged estimate.
  The Kriging estimation is given by:
  (8) $$z^{*}\left(x\right)=\sum_{i=1}^{S}\lambda_{i}\left(x\right)z\left(c_{i}\right),$$
  for each x∈D.
7. Finally, we have an estimation of the values of the target random function $W\left(x\right)=y^{*}\left(x\right)+z\left(x\right)-z^{*}\left(x\right);x\in D$ as the result.

## 4. Results

**Figure 17:**
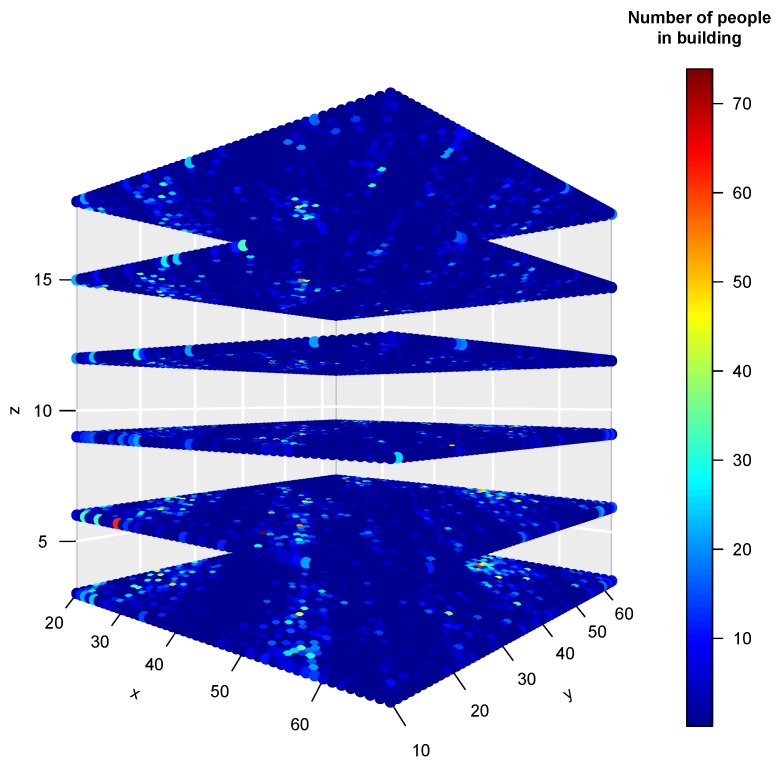
Average 3D scatter plot of NoP in building B4 at WUST campus in 2015.

**Figure 18:**
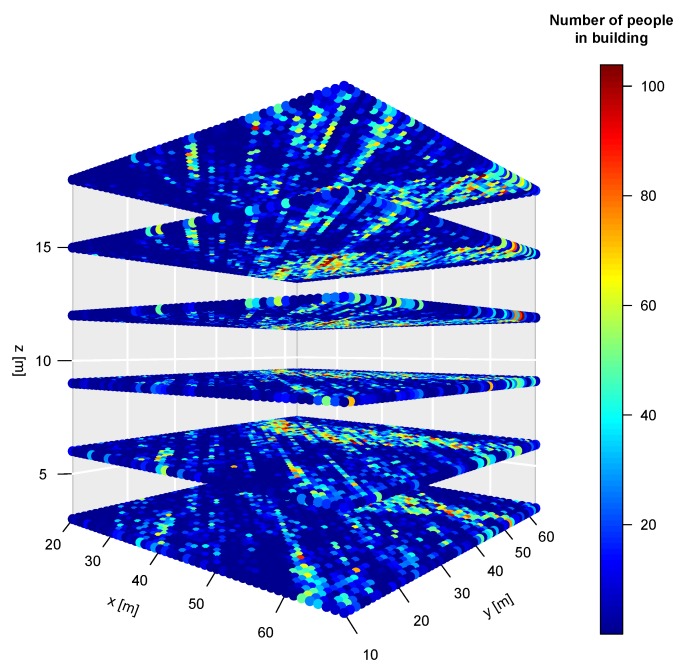
Scatter 3D plot of NoP in WSN in B4 building at WUST in 2016.

We report average results of the geostatistical model computation repeated 100 times using 100 Turning Bands. Results are visualized as a 3D occupancy map of the six levels of building B-4 (ground floor and five stories), where the number of people at each discrete spatial cell is encoded by a hot colormap. Figure 17 and Figure 18 show the occupancy results for 2015 and 2016, respectively. The attendance to the library space is quite unpredictable, and we do not have detailed human-annotated observations of the number of people at each hour interval. It is, however, possible to contrast the geostatistical estimation results with the number of persons in specific offices given the knowledge of the recovered information of control access points, specifically the smart card doors. That way it was possible to assess the occupancy of the offices of the Dean and Vice-Deans of the Faculty of Mechanical Engineering, and the research/teaching staff. Another area where we know the expected NoP is the public attention desk where the library staff will usually be attending the library users. We found a high agreement between the average geostatistical estimations of the NoP in these areas and the actual number of persons extracted from the access control and the administrative information, summarized in Table 2. We have no means to validate the accuracy of the estimations in the general area of the library and halls.

## 5. Discussion

In this section, we give some answers to questions raised by the reviews and some additional clarifications regarding the proposed system and the experimental results.

### 5.1. Effect of Crowd Size and Density

Contrary to artificial vision-based approaches, where high crowd density produces occlusions and therefore hinders the image analysis, our approach is not sensitive to crowd size and density. Increasing crowd density does not produce occlusion effects. However, if the number of access requests is above the AP limit of service, some (many) access requests will be lost, meaning that crowd size sensing is bounded by the APs service capabilities. On the other hand, the approach is highly sensitive to the spatial localization and number of the APs. The Wi-Fi devices will always connect to the closest AP, inducing a Voronoi tessellation of the building space [45]. If this spatial tessellation is not regular, in the sense that some AP covers much more space than other APs, then spatial resolution of the crowd localization will be distorted and biased towards the AP with the large receptive field. The number of APs affects the kriging resolution, consequently higher number of APs allows finer spatial localization of the crowd.

### 5.2. Generalization and Adaptation

Our approach uses the AP activity data recorded during a time interval to carry out the crowd counting and localization process inside the building during this interval. All the computational processes are independent of the time prior to the registered interval. Specifically, kriging needs the Gaussian anamorphosis of the data distribution and the estimation of the covariance function based on the variogram computed on the actual data. They are dependent of the actual spatial distribution of people inside the building, therefore using the estimations from previous time periods would be a serious source of error. In this regard, our approach is radically different from approaches that try to model the signal propagation dependence on the presence of people in the propagation space [9,28,29,30,46], and approaches that build image understanding models from optical sensors, either on the visible [4,10,11,13,17] or infrared [6,23] light range. In these approaches, the data is used to build a predictor of the number of people depending on signal features, so that careful experimental design for training data gathering is required. In our approach, there is no such dependency to training data. On the contrary, using the variogram from previous time intervals corresponding to different spatial localizations of people inside the building would lead to big error in current estimations.

### 5.3. Information Fusion with Other Sensor Networks

The system as illustrated in Figure 1 does not include other sensor information sources, except the physical access control points to some places, such as office doors with smart cards. Our proposed system provides estimations of the localization of people “behind the wall” that can be complementary to information provided by other systems, such as cameras [13] or acoustic sensors [8]. These additional sensors may provide information on people accessing some parts of the building, while our system may provide information on their dispersion or concentration in these building sectors. The most critical issue for such information fusion is the temporal integration of the sensors, because our system may be working in a not so fine temporal resolution. In specific settings where subjects are carrying stress-measuring Wi-Fi enabled wearables [47], the stress information could be fused with the spatial localization to create awareness of potentially dangerous situations. However, these settings may be restricted to very special buildings such as hospitals, or to psychological experiments. One of the risks of this information fusion is the risk associated to the management of personal data.

### 5.4. Privacy Levels

The position that we adopt in this paper is that of ensuring maximal privacy to the subjects [1], therefore our system does not contemplate any identity related information capture or process, not even the MAC of the device which other approaches [2] use for counting and disambiguation purposes. In cases of information fusion personal data protection may be at risk, such as mentioned above. For instance it may be some possibility of linking the face and the stress data via the localization of the person. Differential privacy [48] has not much use in our system, because we are providing de-identified localizations, unless there is a single person in the system. We would propose to remove these limit case localizations when the privacy break may be especially sensitive, such as when dealing with children.

### 5.5. Human Interaction Analysis

Human interaction observation and modeling is a quite interesting topic for research in computer science. It has been studied using visual and depth sensors [49] that allow detailed human motion analysis alone or amid groups. Researchers have been trying to assess intent and the quality of the human communication at diverse levels mostly using cameras [50]. To this endeavor, our system can contribute the detection of hot spots in buildings, i.e., places where people tend to gather for some time to carry out social interactions. Our system is non-intrusive, hence the information would be obtained without perturbing the observed population. On the other hand, it is not possible for us to extract direct social relation links, due to the strict privacy preservation of our approach.

### 5.6. Limitations

The data used in this work is limited to relatively short periods and it is averaged hourly, therefore the results offer low time resolution. Some works in the literature [2] cover only one day, but with greater time resolution and large attendance (over several thousand people). Moreover, the number of people moving around inside the building is not big, so our accuracy claims need further confirmation on buildings with greater attendance, such as buildings devoted to classrooms. We have not tried to identify false individuals (such as Wi-Fi connected printers) using MAC identities, in fact this information was not available to us in the provided logs. We think that this has little effect on the quality of the estimation, because there are few of these devices compared to the people inside the building. Besides, their constant activation and fixed localization would allow correction of the bias introduced by them in the estimation. In the case of disaster areas, our proposed system could be seriously affected when the Wi-Fi infrastructure is down. On the other hand, it can be quickly operative in emergency deployments, provided that the people carry some Wi-Fi enabled device. Another source of concern is the use of corporate devices, such as corporate laptops, which may lead to some redundancy in the correspondence between persons in the crowd and connected devices, in the sense that two different persons may carry the same device at different times. As far as we are counting and estimating the spatial location of persons we are not interested in the specific user identity and this redundancy is irrelevant.

A great limitation of our approach is that it relies in the use of Wi-Fi enabled devices. If people decide to shut down the terminals, for instance by relying only on 4G or 5G connectivity, we lost our source of information. We rely on the fact that users prefer to access Wi-Fi services for several reasons, such as greater bandwidth and contract data limits. Anyway, it can be argued that our approach can be applied to data from 4G or 5G antennas, though with lower spatial resolutions, because each antenna usually covers large spaces when compared to Wi-Fi-APs. However, the access to the data and the design of validation experiences requires deep involvement from mobile phone companies.

## 6. Conclusions

Localization of people in indoor environments while preserving privacy is a challenging problem where several sensing and computational approaches have been reported in the literature. Here we propose the use of already-deployed infrastructure for Wi-Fi services to compute predictive estimations of the number of people inside a building. Using a geostatistical approach to compute spatial occupation estimations it is possible to build a 3D representation of the localization of groups of persons in the building from the measurements of the number of persons accessing the Wi-Fi network through the spatial sparsely distributed access points (APs). In this paper, we have demonstrated the feasibility of the approach on an academic building of non-small size. Estimations cover as far as 60 m and cover six planes, five stories plus the ground floor. Temporal accuracy depends on the temporal resolution of the recorded data. In this demonstration, we had a low temporal resolution, so the estimations made are aggregated in time along the months of the sampling. However, a finer temporal resolution would allow for closer to real-time estimation of the spatial occupation of the building, thus allowing more detailed crowd detection and localization. We claim two advantages of the proposed approach. One is the inherent anonymity of the process, where no personal information is captured or processed in any way. The other is that there is no need for additional infrastructure investment to deploy the system. Future work must be addressed to the extended evaluation of the approach gathering more detailed temporal recordings of the APs as well as the detailed quantitative observation of the actual spatial localization of the people in the building. Research on the modular decomposition [51] of the problem in order to tackle large complex environments is also desirable. 

## Figures and Tables

**Table 1:** Basic statistics of NoP in building B-4 in the two data gathering periods.

Period of Time	Minimum Value	Maximum Value	Mean Value	Standard Deviation	Variability Coefficient
March 2015	0	81	10.61	13.51	127.37
March 2016	0	102	10.68	14.49	135.62

**Table 2:** Accuracy of NoP estimation in specific places of building B-4. D: Dean offices, L: library staff, R: researcher offices.

Accuracy	D	L	R
mean (%)	96	90	95
std. dev.	3.6	4.45	2.3

## Abbreviations

The following abbreviations are used in this manuscript:

APs	Access Points
NoP	Number of People
RSSI	Received signal strength intensity
TB	Turning Bands
WSNs	Wireless Sensor Networks
WUST	WrocÅaw University of Science and Technology
